# Robotic assisted radical nephrectomy with Inferior vena cava tumor thrombus

**DOI:** 10.1590/S1677-5538.IBJU.2023.0245

**Published:** 2023-07-20

**Authors:** Maxwell Sandberg, Wyatt Whitman, Alejandro Rodriguez

**Affiliations:** 1 Wake Forest School of Medicine Medical Center Boulevard Department of Urology Winston Salem NC USA Department of Urology, Wake Forest School of Medicine, Medical Center Boulevard, Winston Salem, NC, USA

## Abstract

**Purpose:**

Surgery for renal cell carcinoma (RCC) with an inferior vena cava (IVC) tumor thrombus can be done via a robotic approach. While this approach is thought to minimize blood loss, it may still result in significant losses (1) and current publications indicate that it can require upwards of 3-day hospital stays (1, 2). However, innovative surgical techniques, such as the split and roll, may curtail this. The purpose of this video is to present the case and surgical technique of robotic assisted radical nephrectomy with IVC thrombectomy.

**Materials and Methods:**

The patient was a 77-year-old male found to have a right upper pole renal mass on CT urogram. On MRI (Figure 1), a renal mass and level II thrombus was seen. For this case, the Da Vinci Xi Intuitive robotic system was used, with four robotic 8-millimeter (mm) metallic trocars, two 5 mm assistant trocars, and one 12 mm air seal port. The split and roll technique were utilized to access the IVC and lumbar veins. This surgical method uses the adventitia of the IVC as a plane of dissection and safely identifies all branches/tributaries of the IVC to minimize the chance of vascular injury (3).

**Results:**

Robotic console time was 150 minutes. The patient had an excellent outcome, with all tumor thrombus removed, less than 50cc of blood loss, and was discharged within 24 hours of the operation. The tumor pathology came back as papillary, high grade, and was stage pT3bN1.

**Conclusions:**

The robotic approach with split and roll technique is a great surgical option for urologists to consider in patients with RCC and a level I or II thrombus, which can minimize blood loss and expedite discharge.

**Figure 1:**
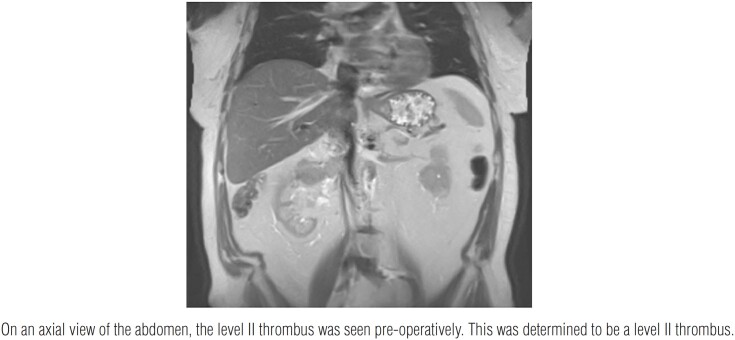
MRI of abdomen.

**Available at:** http://www.intbrazjurol.com.br/video-section/20230245_Rodriguez_et_al

**Int Braz J Urol. 2023; 49 (Video #12): 650-1**
